# The Effect of High-Fat Diet and Exercise Intervention on the TNF-α Level in Rat Spleen

**DOI:** 10.3389/fimmu.2021.671167

**Published:** 2021-12-15

**Authors:** Lin Feng, Feiyun Huang, Yinan Ma, Jialing Tang

**Affiliations:** ^1^ Sport Hospital Affiliated to Chengdu Sport University, Chengdu Sport University, Chengdu, China; ^2^ Key Laboratory of Bio-Resources and Eco-Environment (Ministry of Education), College of Life Sciences, Sichuan University, Chengdu, China; ^3^ Department of Physical Education, Yili Normal University, Yining, China; ^4^ Department of P.E., Central South University, Changsha, China

**Keywords:** high-fat diet, exercise, tumor necrosis factor alpha, spleen, inflammation

## Abstract

High-fat diet (HFD) consumption can trigger chronic inflammation in some tissues. However, it remains unclear if HFD induces chronic inflammation in the spleen. This investigation aims to address the effect of HFD consumption and exercise intervention on the level of tumor necrosis factor alpha (TNF-α) in the spleen. Rats were subjected to HFD feeding and/or moderate-intensity treadmill running. The TNF-α levels in plasma and spleen were detected by ELISA. The mass and total cell numbers of the spleen were measured. In addition, the expression of TNF-α and its relevant gene mRNAs in macrophages from the spleen were analyzed by qRT-PCR. We found that HFD consumption did not significantly affect the mass and total cell numbers of the spleen. However, HFD consumption significantly increased splenic TNF-α level, the expression of TNF-α, toll-like receptor 4, and nuclear factor κB p65 mRNAs. In contrast, the expression of nicotinic acetylcholine receptor alpha 7 subunit (α7nAChR) mRNA in macrophages was downregulated. Additionally, exercise abolished the increase in splenic TNF-α level as well as the abnormal expression of TNF-α and related gene mRNAs in macrophages in HFD-fed rats. In conclusion, our results reveal that HFD consumption increases TNF-α level in the spleen, which is along with upregulation of the expression of TLR4 and NF-κB mRNAs as well as downregulation of the expression of α7nAChR mRNA in splenic macrophages in rats. Exercise abolished detrimental effects of HFD on TNF-α level in the spleen and prevented abnormal expression of these genes in the macrophages from rat spleen.

## Introduction

Excessive consumption of a high-fat diet (HFD) has been well-known to contribute to the onset or development of some metabolic diseases, including obesity, type 2 diabetes mellitus, and insulin resistances (1). The mechanism responsible for detrimental effects of HFD has been studied extensively and appears to be more complex than explanation of energy imbalance (2). Recent studies highlight a crucial role of chronic low-grade inflammatory process in HFD-induced adverse effects (3). It has been shown that HFD consumption not only triggers chronic low-grade inflammation in multiple tissues, including the intestine, liver, adipose tissue, skeletal muscle, and hypothalamus, but also alters homeostasis in these tissues (3).

The spleen is an important immune organ where most of immune responses initiate and is responsible for the production of most of inflammatory cytokines in rodents and humans (4). In addition, the spleen preserves an anti-inflammatory immune environment and is involved in the modulation of immune balance (4). In the context of lipopolysaccharide-induced endotoxemia, the spleen releases newly synthesized tumor necrosis factor alpha (TNF-α) into the liver *via* splenic vein; and from there, it enters into systemic circulation, and it is also the primary source of systemic TNF-α in endotoxemia (5). TNF-α is a vital cytokine, which can generate many deleterious effects, such as inducing other pro-inflammatory cytokines and promoting macrophage infiltration (5). However, in the context of excessive consumption of HFD, it is not clear whether HFD triggers TNF-α increase in the spleen. Given that both endotoxin lipopolysaccharide and fatty acids target common receptor [toll-like receptor 4 (TLR4)] (6, 7), we reason that excessive consumption of HFD may promote TNF-α production in the spleen. Therefore, this investigation aims to analyze the effect of HFD consumption on TNF-α level in the plasma and the spleen. Because physical exercise can reduce low-grade inflammation and prevent or improve obesity, diabetes, and dyslipidemia (8), we also evaluate the role of exercise intervention in this process. The results achieved may shed new light on the management of HFD consumption-associated diseases.

## Materials and Methods

### Animal and Protocols

Male Sprague–Dawley rats weighting 200 ± 10g (Huafukang Co., Peking, China) were used in this study. The rats were housed in individual plastic cages with free access to water under the environment with constant temperature (23°C ± 2°C), humidity (45%–55%), and 12-h light/dark cycle. Animal studies were approved by the Animal Utilization Committee of Sport Hospital Affiliated to Chengdu Sport University and conducted under NIH Guidance for the Care and Use of Laboratory Animals.

Rats were divided into four groups: HFD group (n = 8), HFD+exercise group (n = 8), standard diet control group (n = 8), and standard diet+exercise group (n = 8). HFD and standard diet control groups were randomly assigned except for the exercise groups. Random numbers were generated using a computer-based random-order generator. Exercise groups were chosen basing on whether rats are adaptable to treadmill running. Rats in the HFD and HFD with exercise groups were provided with an HFD (60% kcal fat; Huafukang Co., Peking, China), while the rats in the control group and standard diet+exercise group were provided with standard laboratory chow (17% kcal fat; Huafukang Co., Peking, China) for 4 weeks. Exercise-trained groups (HFD+exercise group and standard diet+exercise group) underwent treadmill running exercise during HFD feeding (4 weeks). Rats that died, that did not finish exercise, or with data outliers were excluded. After 4 weeks, body weight of rats in each group was measured, and blood samples were collected from the retro-orbital sinuses into Eppendorf tubes containing EDTA, and plasma was prepared and frozen at −80°C. Then, the rats were sacrificed by decapitation, and the spleen tissue was taken and weighed after blotting dry with filter paper. Splenocytes and splenic macrophages were isolated.

### Exercise Regimen

Treadmill running was utilized for exercise intervention. To identify which rats were enrolled in the exercised groups, all rats were subjected to assimilation training for 2 weeks prior to the start of the experiment. Assimilation training was conducted for 30 min every time, twice a week, on a motorized treadmill, starting with a 10-min running warm-up at the speed of 6 m/min, followed by acceleration to 18 m/min gradually. Rats adaptable to treadmill running were chosen for the exercised group.

During exercise sessions, the exercise began with 10-min warm-up at 6 m/min and then followed by a given running that sets at 8 m/min. Then the speed was increased to 18 m/min on a motorized five-lane treadmill at 0° incline for moderate-intensity exercise. Running exercise was performed 30 min/day, 5 days/week, for four consecutive weeks.

### Splenocyte and Splenic Macrophage Isolation

Splenocytes and macrophages were isolated from rats in each group as described by Lahat (9). In brief, the whole spleen was pressed with a syringe plunger through a 40-G stainless steel screen in Roswell Park Memorial Institute (RPMI) 1640 culture medium (HyClone, Logan, USA). The cell suspension was centrifuged at 1,000*g* for 5 min at 4°C. Erythrocytes were lysed with distilled water to obtain splenocytes. Then, the splenocytes were washed and were suspended in RPMI 1640 medium. The cell number was counted using a hemocytometer. Subsequently, 10^7^ cells were cultured in RPMI 1640 medium, supplemented with 20% heat-inactivated fetal calf serum (Gibco, USA), penicillin, and streptomycin (Biological Industries, Israel) for 90 min. Non-adherent cells were removed, and the adherent cells were centrifuged and harvested to obtain a macrophage-rich cell preparation. The enrichment of macrophages was examined by fluorescence-activated cell sorting (FACS) using Flow Cytometer (BD Fortessa; BD Biosciences, San Jose, CA, USA) and FlowJo software (FlowJo, Ashland, OR, USA) after macrophages were labeled with fluorescein isothiocyanate (FITC)-anti-rat CD11b/c monoclonal antibody (OX42; BD PharMingen, San Diego, CA, USA). Results are presented as mean of fluorescence intensity.

### ELISA Analysis

TNF-α level was measured using a commercially available enzyme-linked immunosorbent assay kit (Lianke, Hangzhou, China) following the manufacturer’s instructions. Protein concentrations were quantified using bicinchoninic acid protein assay kit (MLBIO Biotechnology Co. Shanghai, China).

### Quantitative Real-Time PCR

For gene expression analysis, total RNA from splenic macrophages was extracted using TRIzol (Solarbio Co., Beijing, China) according to manufacturer’s instructions. RNA concentration and purity were examined by an ultraviolet spectrophotometer. cDNAs were synthesized with a cDNA synthesis kit (Solarbio Co., Beijing, China). The mRNA levels of TNF-α, TLR4, nuclear factor κB p65, nicotinic acetylcholine receptor alpha 7 subunit (α7nAChR), and β-actin were measured using a SYBR Green PCR mix (Solarbio Co., Beijing, China) on an Rotor-Gene Q (Qiagen, Germany). The primer sequences were designed using Primer Express (version 2.0.0), and the primers were synthesized by Shanghai Generay Biological Engineering Co., Ltd. All primer sequences are listed in **Table 1**. The relative levels of target gene expression were calculated using the Ct method and normalized to the expression of the housekeeping gene β-actin.

**Table 1:** The sequences of primers used for qRT-PCR.

Target gene	Forward	Reverse
TNF-α	GCCTCTTCTCATTCCTGCTT	TGGGAACTTCTCATCCCTTTG
TLR4	CCGCTCTGGCATCATCTTCA	CCCACTCGAGGTAGGTGTTTCTG
NF-κB	GTGGGCAAGCACTGTGAGGA	TCATCCGTGCTTCCAGTGTTTC
α7nAChR	CTCATGGGAATCCCTGGCAAA	GAGCCAGGGCTGAAATGAGT
β-Actin	GGTGGGGCGCCC CAGGCACCA	GCTCCTTAATGT CACGCACGA

### Statistical Analysis

Data were expressed as mean ± SD. Data were analyzed by Student’s t-tests of unpaired samples for the statistical difference between groups or one-way analysis of variance (ANOVA) followed by Tukey’s test for multiple groups using the GraphPad Prism 5.0 software. p < 0.05 was regarded as a statistically significant difference.

## Results

### The Effect of HFD on Rat Body Weight, Spleen Mass, and Splenic TNF-α Level

**Figure 1:**
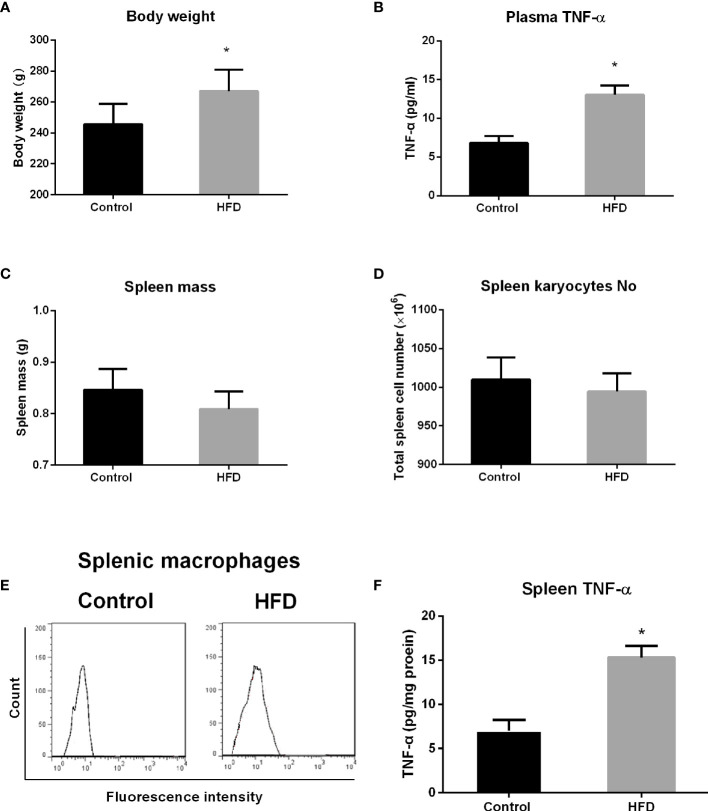
The effect of high-fat diet (HFD) consumption on body weight, spleen mass, and tumor necrosis factor alpha (TNF-α) level in the spleen from rats. After rats consumed HFD for 4 weeks, body weight, spleen mass, total spleen cell number, splenic macrophage enrichment, and TNF-α level in the plasma and the spleen in control and HFD groups were analyzed. **(A)** Body weight; **(B)** TNF-α level in the plasma; **(C)** spleen mass; **(D)** total spleen cell number; **(E)** fluorescence-activated cell sorting (FACS) histograms of splenic macrophages; and **(F)** TNF-α level in the spleen. Values are mean ± SD. n = 8. Data were analyzed by Student’s t-tests of unpaired samples. *p < 0.05 *vs*. control.

Prior to the experiment, the rats in the control and HFD groups had similar body weights. Following consumption of HFD for 4 weeks, the rats in the HFD group had significant increase in body weight compared with that in the control group (p = 0.0071) (**Figure 1A**). Blood samples were collected to detect TNF-α level by ELISA. Consistent with other studies (1), HFD-fed rats showed higher TNF-α level in the plasma compared with standard chow-fed control rats (p < 0.0001) (**Figure 1B**). Spleen was obtained after rats were sacrificed. Spleen mass, total cell number, macrophage enrichment, and TNF-α level in the spleen were analyzed. Although the spleen mass and total spleen cell number decreased in the HFD group, there is no statistical significance compared with control groups (**Figures 1C, D**). Also, no statistical difference in macrophage enrichment was detected in these two groups (**Figure 1E**). However, the spleen TNF-α level was significantly elevated in HFD-fed rats compared with the control group (p < 0.0001) (**Figure 1F**).

### The Effect of HFD on the Expression of TNF-α and Its Relevant Genes in Macrophages from the Spleen

**Figure 2:**
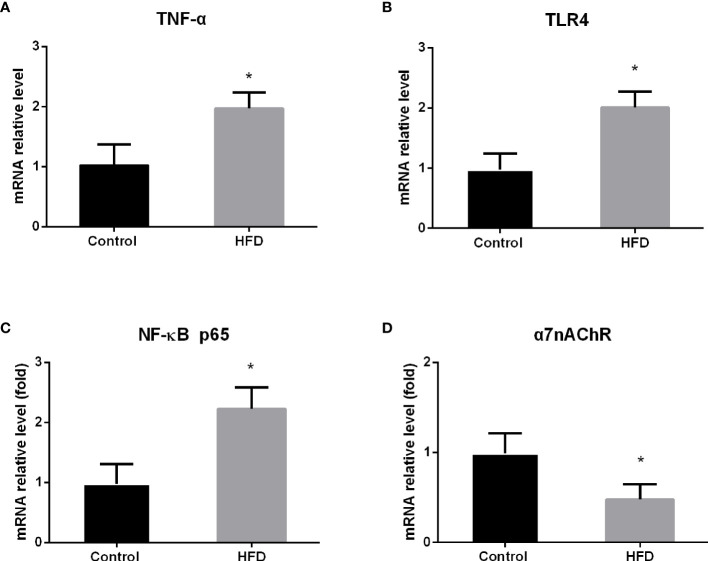
The effect of high-fat diet (HFD) consumption on the expression of TNF-α and the relevant genes in splenic macrophages. After HFD consumption for 4 weeks, macrophages were isolated from rat spleen. Then, the expression of tumor necrosis factor alpha (TNF-α) and its related genes was analyzed by qRT-PCR. **(A)** TNF-α mRNA expression level; **(B)** toll-like receptor 4 (TLR4) mRNA expression level; **(C)** nuclear factor κB (NF-κB) p65 mRNA expression level; and **(D)** nicotinic acetylcholine receptor alpha 7 subunit (α7nAChR) mRNA expression level. Values are mean ± SD. Data were analyzed by Student’s t-tests of unpaired samples. *p < 0.05 *vs*. control.

Given that macrophages are a major source of TNF-α release in the spleen (10), we isolated macrophages from the spleen to further evaluate the expression of TNF-α by qRT-PCR. We found that HFD consumption significantly increased the expression of TNF-α mRNA in splenic macrophages, and significant difference in the level of TNF-α expression was detected between HFD and control groups (p < 0.0001) (**Figure 2A**), suggesting that HFD consumption results in low-grade inflammation in the spleen.

It has been shown that TLR4 mediates free fatty acids and lipopolysaccharide-associated inflammation and that nuclear factor κB (NF-κB) is a pivotal signal molecule for TNF-α production (11, 12). To understand the mechanism by which HFD increases TNF-α expression, we analyzed the expression of TLR4 and NF-κB p65 mRNA in splenic macrophages by qRT-PCR. Consistent with the alteration of TNF-α mRNA, the mRNA levels of TLR4 and NF-κB p65 were significantly elevated in splenic macrophages from HFD rats, compared with those from control rats (p < 0.0001; p < 0.0001) (**Figures 2B, C**). This result suggests that HFD intake increases TNF-α production through activating TLR4/NF-κB signaling pathway in splenic macrophages.

Macrophages in the spleen also express α7nAChR, which is an essential component in the vagus nerve-based cholinergic anti-inflammation pathway (13). Upon activation, α7nAChR suppresses TNF-α production and release in splenic macrophages (14, 15). To further understand the effect of HFD on TNF-α production, we analyzed the level of α7nAChR mRNA in splenic macrophages by qRT-PCR. In contrast to the change of TLR4 expression, we found that the level of α7nAChR mRNA was significantly downregulated in splenic macrophages from HFD-fed rats compared with control rats (p = 0.001) (**Figure 2D**), suggesting the reduction of anti-inflammatory capacity in the spleen in HFD-fed rats.

### The Effect of Exercise Intervention on the Expression of TNF-α and Its Relevant Genes in Splenic Macrophages in HFD-Fed Rats

**Figure 3:**
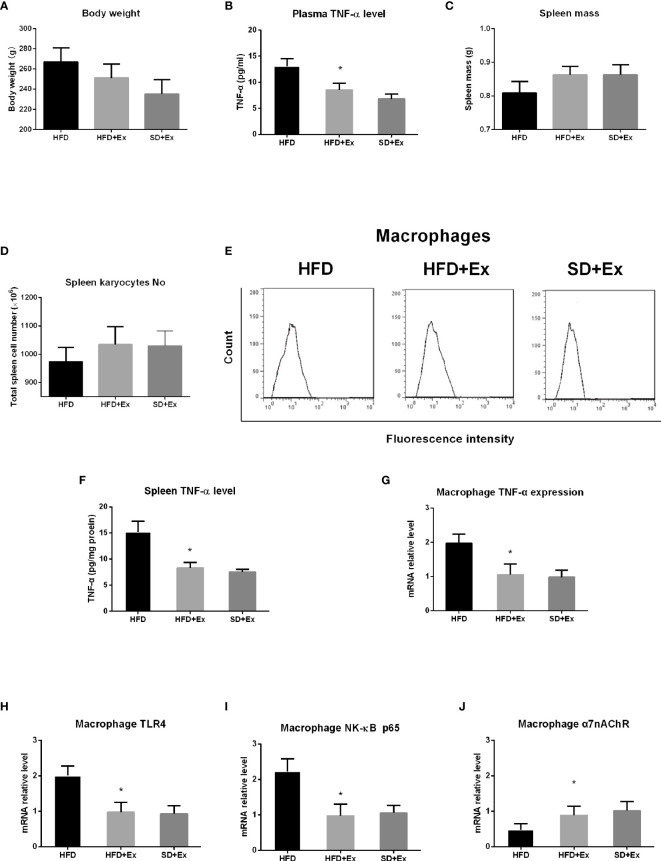
The effect of exercise intervention on TNF-α in the spleen from rats with high-fat diet (HFD) feeding. Rats were subjected to moderate-intensity running exercise during HFD feeding. After 4 weeks, body weight, spleen mass, total spleen cell number, and TNF-α level in the plasma and the spleen were measured in HFD group with exercise (HFD+Ex group), HFD group without exercise (HFD group), and standard diet group with exercise (SD+Ex). Macrophages were isolated from the spleen in rats from these groups to analyze the expression of TNF-α and its related genes by qRT-PCR. **(A)** Body weight; **(B)** TNF-α level in the plasma; **(C)** spleen mass; **(D)** total spleen cell number; **(E)** fluorescence-activated cell sorting (FACS) histograms of splenic macrophages; **(F)** TNF-α level in the spleen; **(G)** the expression level of TNF-α mRNA in splenic macrophages; **(H)** the expression level of TLR4 mRNA in splenic macrophages; **(I)** the expression of NF-κB p65 mRNA in splenic macrophages; and **(J)** the expression of α7nAChR mRNA in splenic macrophages. Values are mean ± SD. Data were analyzed by one-way analysis of variance (ANOVA) followed by Tukey’s test. *p < 0.05 *vs*. HFD group.

To evaluate the effect of exercise on TNF-α level in the plasma and the spleen, a group of rats was subjected to moderate-intensity running exercise on treadmill during HFD feeding, which was compared with the standard diet group with exercise (SD+Ex) and the HFD group without exercise (HFD group). After 4 weeks, body weight decreased in HFD rats with exercise (HFD+Ex group), but it is not statistically significant compared with the HFD group (**Figure 3A**). TNF-α level in the plasma was measured by ELISA. We found that TNF-α levels in the plasma reduced significantly in HFD+Ex group compared with the HFD group (p < 0.0001) (**Figure 3B**). Interestingly, there was no statistically significant difference for TNF-α levels between the SD+Ex group and HFD+Ex group (**Figure 3B**).

Next, we analyzed spleen mass, total spleen cell number, macrophage enrichment, and splenic TNF-α level in the three groups (HFD, HFD+Ex, and SD+Ex groups). The rats in the HFD+Ex group showed increase in spleen mass and total spleen cell number, but the difference was not significant compared with that in the SD+Ex group (**Figures 3C, D**). No significant difference in macrophage enrichment was noticed in the three groups (**Figure 3E**). However, the splenic TNF-α levels decreased significantly in the HFD+Ex group compared with the HFD group, although no significant difference in splenic TNF-α levels was detected between the HFD+Ex and SD+Ex groups (**Figure 3F**).

Finally, macrophages were isolated from the spleen to analyze the expression of TNF-α, TLR4, NF-κB p65, and α7nAChR genes by qRT-PCR. We found that there was a significant lower level of TNF-α mRNA in splenic macrophages in HFD+Ex group than in the HFD group (p < 0.0001) (**Figure 3G**). Concomitantly, the levels of TLR4 and NF-κB p65 mRNA were significantly downregulated in the HFD+Ex group compared with the HFD group (p = 0.0003; p < 0.0001) (**Figures 3H, I**); the expression of α7nAChR mRNA was significantly elevated following exercise, and the difference was statistically significant between the HFD+Ex and HFD groups (p = 0.0027) (**Figure 3J**). However, between the SD+Ex group and HFD+Ex group, no statistically significant difference was detected for the expression of these genes in splenic macrophages. These results indicate that moderate-intensity exercise inhibits HFD-induced increase of TNF-α levels in system and the spleen and prevents abnormal expression of TNF-α, TLR4, NF-κB, and α7nAChR mRNAs in splenic macrophages.

## Discussion

In the current study, we found that 4-week HFD consumption significantly increased TNF-α level in rat spleen, which was accompanied by upregulating the mRNA levels of TNF-α, TLR4, and NF-κB p65, and by downregulating the mRNA of α7nAChR in macrophages from rat spleen. This result suggests that the effect of HFD on TNF-α level in the spleen may be attributed to impaired balance of inflammation and anti-inflammation in the spleen. Additionally, we demonstrated that moderate-intensity exercise during HFD feeding abolished the detrimental effect of HFD on TNF-α level in the spleen and prevented abnormal expression of TNF-α and other relevant genes in splenic macrophages.

Previous studies have verified that HFD results in increased expression or production of TNF-α in intestinal macrophages (16), adipose tissue macrophages (17), hypothalamic microglia (the central nervous system counterpart of macrophages) (18), peritoneal macrophages (19), and hepatic Kupffer cells (20). In the current study, we found that 4-week HFD consumption significantly increased TNF-α expression in splenic macrophages from rats. It appears that elevated TNF-α expression in macrophages might play an important role in HFD-associated increase in splenic TNF-α level. Indeed, macrophage depletion has been shown to suppress the infiltration of macrophages in some tissues and the production of inflammatory cytokines (8). TNF-α is mainly released from activated macrophages and can trigger a deleterious signaling cascade to induce the production of other pro-inflammatory cytokine (21), and the increase of macrophage infiltration (22). Based on the crucial role of TNF-α in chronic low-grade inflammation, it has been used as markers to monitor the dynamic change of inflammation in responses to high-fat meals and exercise (23).

In the current study, we also demonstrated significant increase in plasma TNF-α in HFD rats. High levels of circulating TNF-α are believed to lead to severe inflammatory response, metabolic alteration, and insulin resistance (24, 25). In lipopolysaccharide-induced endotoxemia, the spleen has been verified to be the major source of circulating TNF-α (5). In the context of HFD feeding, elevated circulating TNF-α may come from different tissues, such as the spleen and white adipose tissue, because of a change in the inflammatory profile in multiple tissues. The white adipose tissue has been shown to release TNF-α and other inflammatory mediators into the circulation (24).

We found that increased TNF-α level in the spleen is accompanied with upregulation of TNF-α, TLR4, NF-κB mRNAs, and downregulation of α7nAChR mRNA. TLR4 is one of the toll-like receptors, a family of proteins playing a role in the innate immune system, and is believed to be an important trigger of obesity-associated inflammatory response (26). Saturated fatty acids and lipopolysaccharides are agonists for TLR4, which can bind to and activate TLR4 signaling pathways, subsequent transcription factor NF-κB, to lead to production of pro-inflammatory cytokines, including TNF-α (26). α7nAChR was identified as a major component of efferent vagus nerve-based cholinergic anti-inflammatory pathway (13). This pathway is a predominant modulatory circuitry in neural regulation of immunity and inflammation, and it interacts with immune cells to modulate and restrain chronic inflammation (27). Activation of this pathway improves blood glucose, insulin resistance, and other obesity-associated complications in mice fed with HFD (28). α7nAChR activation suppresses pro-inflammatory cytokine production and release by inhibition of NF-κB in splenic macrophages (14). *In vitro*, antigen-stimulated spleen cells from α7nAChR-deficient mice produce more TNF-α (29). In the current study, the upregulation of TLR4 and NF-κB genes and downregulation of α7nAChR expression by HFD consumption suggest it tips the balance of TNF-α regulation in splenic macrophages. It also suggests that inflammatory response increases but anti-inflammatory capacity decreases in the spleen, which is in agreement with the notion that disruption of immune homeostasis is a key aspect of low-grade inflammation development (4). Specifically, reduction of anti-inflammatory capacity in the spleen, as demonstrated by downregulation of α7nAChR expression in macrophages, is likely more critical for HFD-induced low-grade inflammation because vagus nerve regulation of peripheral anti-inflammatory activity mainly depends on the spleen (4).

It has been shown that HFD can result in splenic lesions, such as histological changes, atrophy, splenocyte apoptosis, and lipotoxicity (30, 31). In the current study, we observed that HFD decreased spleen mass and total spleen cell number, although the decrease is not statistically significant. However, HFD did not affect splenic macrophage number. The change of spleen mass and total spleen cell number may indicate splenocyte apoptosis. Inflammatory factors are shown to trigger splenocyte apoptosis (32). In our study, HFD-induced increase in splenic TNF-α level might be a major cause for the change of spleen mass and total spleen cell number.

In the present study, we also found that moderate-intensity running exercise for 4 weeks abolished not only the decrease in spleen mass and total spleen cell number but also the increase of TNF-α expression in splenic macrophages during HFD feeding. Physical exercise, an inexpensive and side effects-free way, has been well proven to be an effective clinical intervention to reduce body weight and improve insulin resistance and type 2 diabetes mellitus (33, 34). Exercise also decreased HFD-induced body weight gain and metabolic syndrome in experimental rats (35). The beneficial effects of physical exercise on obesity and type 2 diabetes mellitus have been verified to be involved in the decrease in chronic low-level inflammation (19, 34). Indeed, physical exercise is documented to markedly restrain chronic low-grade inflammation and is acknowledged as an efficient anti-inflammatory intervention (8, 36, 37). However, the molecular mechanism about its beneficial effects is poorly understood. One study shows that voluntary wheel-running exercise inhibits the increase of TNF-α expression in peritoneal macrophages in mice (19). In another study targeting macrophages, suppression of TNF-α production has been shown to be the basis of mechanical stress anti-inflammatory effect (38). A meta-analysis including 17 animal studies concludes that chronic endurance exercise leads to a marked tendency towards TLR4 downregulation in macrophages and other immune cells in rodents with obesity or metabolic syndrome (39). Based on the fact that physical exercise enhances cardiac parasympathetic tone, Lujan hypothesizes that α7nAChR cholinergic anti-inflammatory pathway mediates the anti-inflammatory phenotype associated with physical exercise (36). Similarly, through analyzing the effect of exercise on the levels of brain-derived neurotrophic factor that increases cholinergic activity, Papathanassoglou also proposed that physical exercise likely upregulates α7nAChR signaling in the central and peripheral nervous system and in immune cells (40). Intriguingly, in the present study, running exercise downregulates the expression of TLR4 and NF-κB mRNA and meanwhile upregulates the expression of α7nAChR mRNA in splenic macrophages from HFD feeding rats, suggesting that moderate-intensity physical exercise maintains the balance of TNF-α production in splenic macrophages, or the balance of inflammatory and anti-inflammatory activities in the spleen during HFD feeding.

It is clear that the efficacy of exercise depends on its duration, intensity, and modality (41). A minimum of 150 min of moderate-to-vigorous intensive physical activity per week is able to promote health, as recommend by the American College of Sports Medicine (42). Previous studies show that, in humans and in animal models, 2 weeks’ moderate-intensity exercise can lower blood glucose level and improve plasma lipoprotein profiles (43). In the current study, it is not surprising that moderate-intensity running exercise for four consecutive weeks prevents TNF-α expression in splenic macrophages during HFD feeding.

Taken together, our results reveal that HFD consumption leads to increase in TNF-α level in the spleen, which is along with upregulation of TLR4 and NF-κB expression, as well as downregulation of α7nAChR expression in splenic macrophages from rats. Exercise reduced TNF-α level in the spleen and prevented abnormal expression of TNF-α and its relevant genes in macrophages in HFD-fed rats. Therefore, this research may deepen our understanding of the pathogenesis of HFD-associated diseases and shed light on the management of these diseases.

## Data Availability Statement

The raw data supporting the conclusions of this article will be made available by the authors, without undue reservation.

## Ethics Statement

The animal study was reviewed and approved by the Ethics Committee of Hebei North University.

## Author Contributions

LF, FH, and YM contributed to the conception of the study; performed the experiment; LF and FH contributed significantly to the analysis and manuscript preparation; LF, FH, YM and TJ performed the data analyses and wrote the manuscript. All authors contributed to the article and approved the submitted version.

## Conflict of Interest

The authors declare that the research was conducted in the absence of any commercial or financial relationships that could be construed as a potential conflict of interest.

## Publisher’s Note

All claims expressed in this article are solely those of the authors and do not necessarily represent those of their affiliated organizations, or those of the publisher, the editors and the reviewers. Any product that may be evaluated in this article, or claim that may be made by its manufacturer, is not guaranteed or endorsed by the publisher.
