# Corrigendum: The Effect of High-Diet and Exercise Intervention on the TNF-α Level in Rat Spleen

**DOI:** 10.3389/fimmu.2022.880142

**Published:** 2022-03-14

**Authors:** Lin Feng, Feiyun Huang, Yinan Ma, Jialing Tang

**Affiliations:** ^1^ Sport Hospital Affiliated to Chengdu Sport University, Chengdu Sport University, Chengdu, China; ^2^ Key Laboratory of Bio-Resources and Eco-Environment (Ministry of Education), College of Life Sciences, Sichuan University, Chengdu, China; ^3^ Department of Physical Education, Yili Normal University, Yining, China; ^4^ Department of P.E., Central South University, Changsha, China

**Keywords:** high-fat diet, Exercise, tumor necrosis factor alpha, spleen, inflammation

Co-authors Feiyun Huang and Jialing Tang were unintentionally omitted as authors in the original published article. The corrected author list and Author Contribution Statement appears below.

The authors apologize for this error and state that this does not change the scientific conclusions of the article in any way. The original article has been updated.

Lin Feng^1†^, Feiyun Huang^2†^, Yinan Ma^3*^, Jialing Tang^4*^

^1^Sport Hospital Affiliated to Chengdu Sport University, Chengdu Sport University, Chengdu, China

^2^Key Laboratory of Bio-Resources and Eco-Environment (Ministry of Education), College of Life Sciences, Sichuan University, Chengdu, China

^3^Department of Physical Education, Yili Normal University, Yining, China

^4^Department of P.E., Central South University, Changsha, China

## Author Contribution

LF, FH, and YM contributed to the conception of the study; performed the experiment; LF and FH contributed significantly to the analysis and manuscript preparation; LF, FH, YM and TJ performed the data analyses and wrote the manuscript. All authors contributed to the article and approved the submitted version.

## Publisher’s Note

All claims expressed in this article are solely those of the authors and do not necessarily represent those of their affiliated organizations, or those of the publisher, the editors and the reviewers. Any product that may be evaluated in this article, or claim that may be made by its manufacturer, is not guaranteed or endorsed by the publisher.

